# Ancient but Efficient: Two Case Reports of Pedicle-Tubed Groin Flap for Soft Tissue Coverage in Upper Limb Defects

**DOI:** 10.7759/cureus.23610

**Published:** 2022-03-29

**Authors:** Mohd Farid Mohd Amin, Shawaltul Akhma Harun Nor Rashid, Wan Azman Wan Sulaiman, Muath Mamdouh Mahmod Al-Chalabi

**Affiliations:** 1 Reconstructive Sciences Unit, Universiti Sains Malaysia (USM), Kota Bharu, MYS; 2 Plastic and Reconstructive Surgery, Hospital Raja Perempuan Zainab II, Kota Bharu, MYS

**Keywords:** soft tissue coverage, tube flap, pedicled tubed groin flap, distal flap, groin flap

## Abstract

The distant tubed pedicle skin flap concept was introduced almost 100 years ago. It was previously considered the workhorse flap to reconstruct the face and upper limbs. Despite being considered an ancient reconstruction method, it is still widely used by many reconstructive surgeons. It is considered a versatile flap with a good outcome. This article presents two cases of soft tissue injuries in the upper limbs reconstructed using distant tubed pedicled groin flaps, one of the types of tubed pedicle flaps. This article highlights the relevance of the tubed distant pedicle flap in reconstructing the upper limb defects and its effectiveness in restoring function, even though the cosmetic outcome is not favorable.

## Introduction

McGregor and Jackson first proposed the tubed pedicle groin flap in 1972 [1]. He used the concept of the tubed distant pedicle flap that Gillies, Filatov, and Ganzer introduced in 1917, where they used method to reconstruct the significant skin defects during World War I [1,2]. The pedicled groin flap is a reliable flap with axial blood flow. It has been used to reconstruct acute traumatic complex defects and traumatic hand and forearm defects since the 1970s and 1980s [1]. Despite the development of microvascular surgery, pedicled groin flap is still being practiced, especially in high-risk patients and with technical difficulties [3]. The groin flap is based on the superficial circumflex iliac artery (SCIA). It arises from the femoral artery 2 cm below the inguinal ligament, then crosses the sartorius muscle and runs laterally toward the anterior superior iliac spine. Groin flaps are designed along the course of the SCIA [4]. This paper presents two cases of the upper extremity soft-tissue defects, which were reconstructed with the tubed pedicled groin flaps.

## Case presentation

Case 1

**Figure 1:**
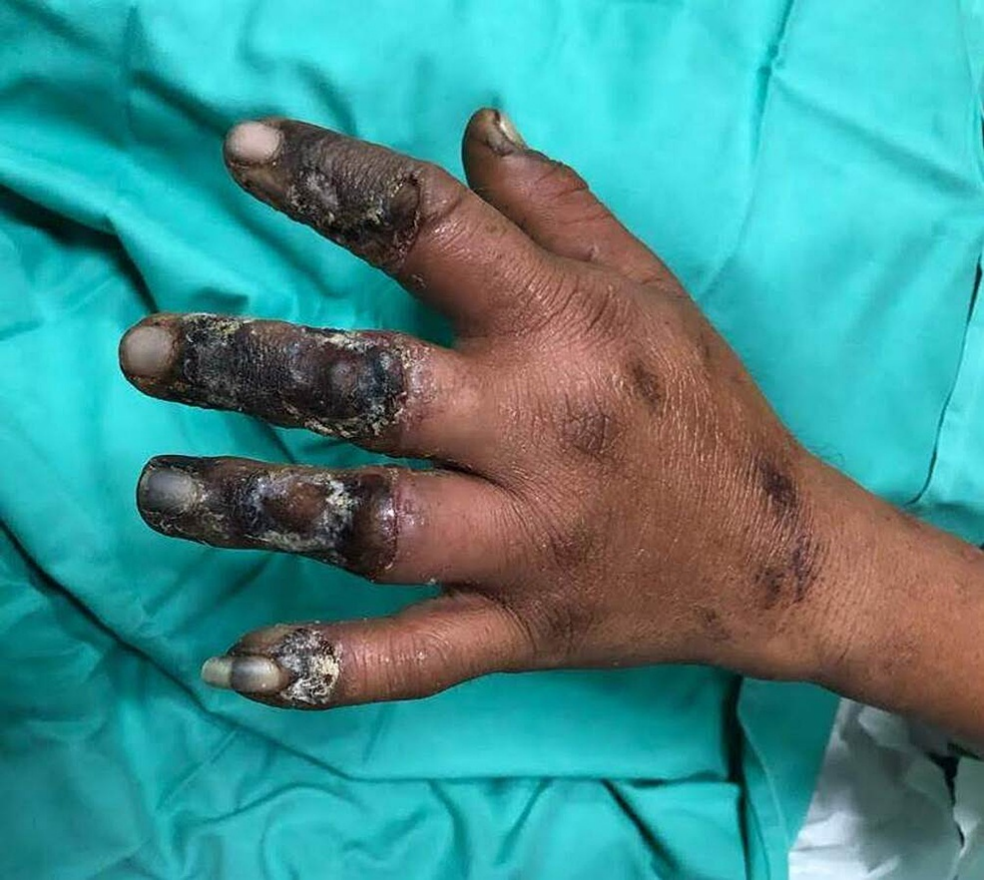
A full-thickness burn injury involving the dorsal aspect of the left second to fifth fingers.

A 33-year-old gentleman was referred to our center, where he was involved in an industrial injury. The left hand was crushed under a piece of huge wood. It was in contact with the hot chainsaw metal for more than one minute. A full-thickness burn injury was noted on a presentation involving the dorsal aspect of the left second to fifth fingers, as shown in Figure 1.

**Figure 2:**
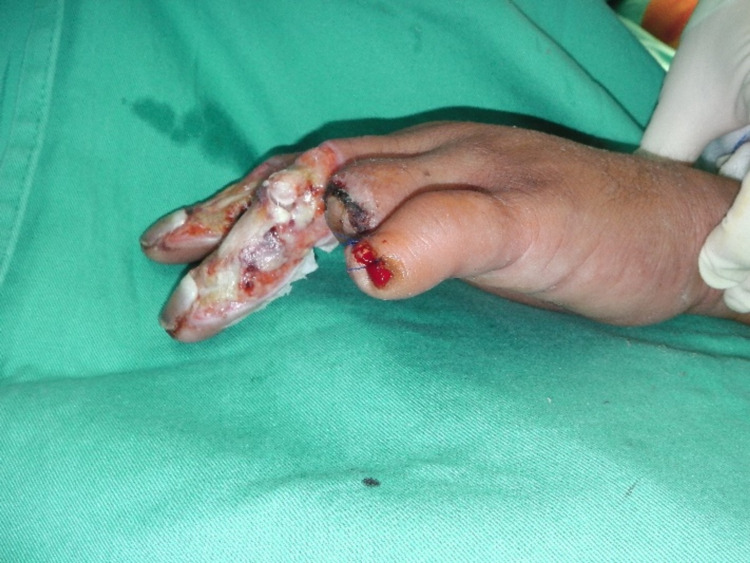
Post-wound debridement of the index and middle fingers and post-amputation of the ring and little fingers.

The wounds were exposing the distal phalanges of the second to fourth fingers. Evaluation at one-week post-injury noted the ring and little fingers were gangrenous. The exposed bones were desiccated and necrosed; hence, they were amputated at the level of the neck of the phalanx and distal interphalangeal joint, respectively. The left middle and index fingers were debrided on the dorsal part, exposing the external tendons and bones that are still healthy. The wound was almost circumferential post-debridement with only a small area of intact skin on the radial side, as shown in Figure 2.

**Figure 3:**
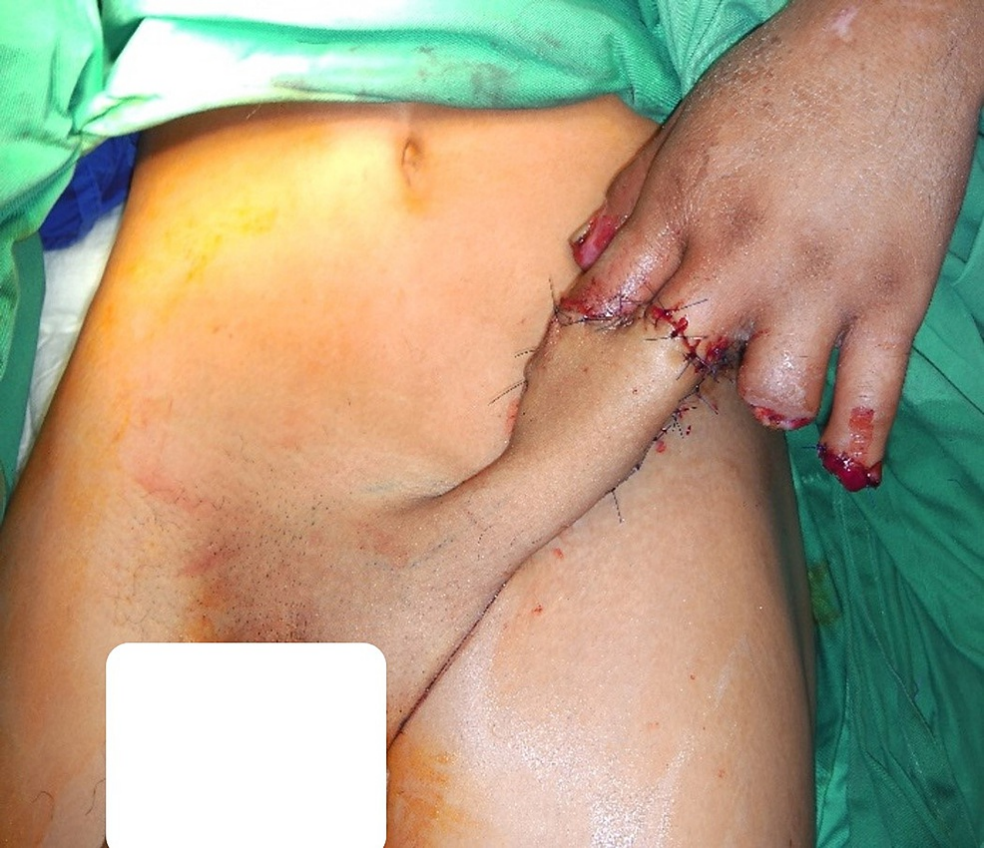
Pedicled groin flap was tubed and enveloped the defects of the index and middle fingers.

The left pedicled groin fasciocutaneous flap was designed based on the ipsilateral superficial iliac circumflex artery and raised from laterally to medially. The lateral part of the donor site closed primarily, leaving the fasciocutaneous flap with the pedicle within. The flap enveloped the left middle and index fingers, making a tube appearance in Figure 3.

**Figure 4:**
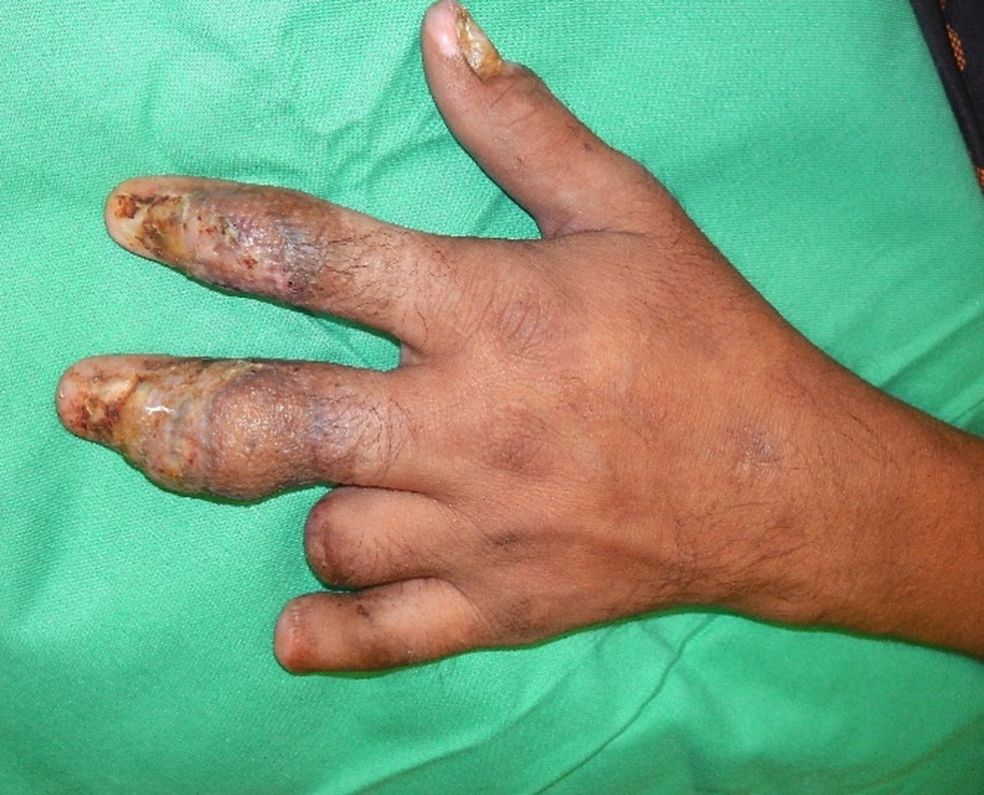
The appearance of the index and middle finger two months after the operation.

Post-operation, the left arm was adducted to the left groin, and the flap was divided after 21 days. Because of the thick and bulky flap, it was thinned and subsequently grafted with a full-thickness skin graft harvested from the right inguinal area. Figure 4 shows the appearance of the left middle and index fingers two months post-operation.

Case 2

**Figure 5:**
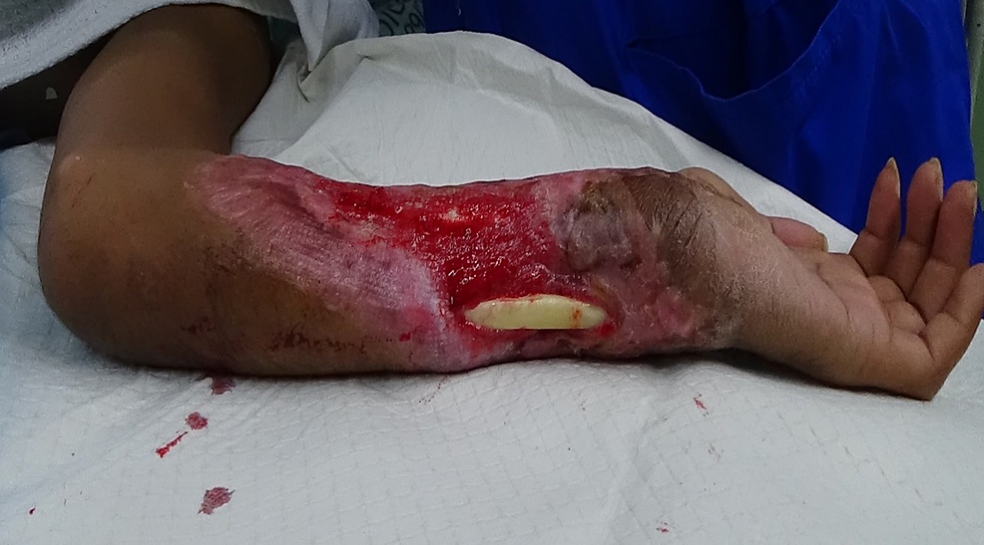
Wound over the anteromedial aspect of the distal left forearm containing exposed distal part of the left ulnar bone with a length of 7 cm and presence of pink granulation tissue.

A 24-year-old gentleman was involved in an industrial-related injury. His left upper limb was in contact with hot steel for almost an hour, leading to a full-thickness burn on the left forearm with compartment syndrome. He underwent an emergency fasciotomy and multiple subsequent wound debridements for an infected fasciotomy wound. Because of that, he had a significant wound on his left forearm with an exposed left ulnar bone. The wound was on the anteromedial aspect of the distal left forearm and measured 11 x 4 cm with the presence of pink granulation tissue. Also, there was an exposed distal part of the left ulnar bone with a length of 7 cm, but the bone was still viable, as shown in Figure 5.

**Figure 6:**
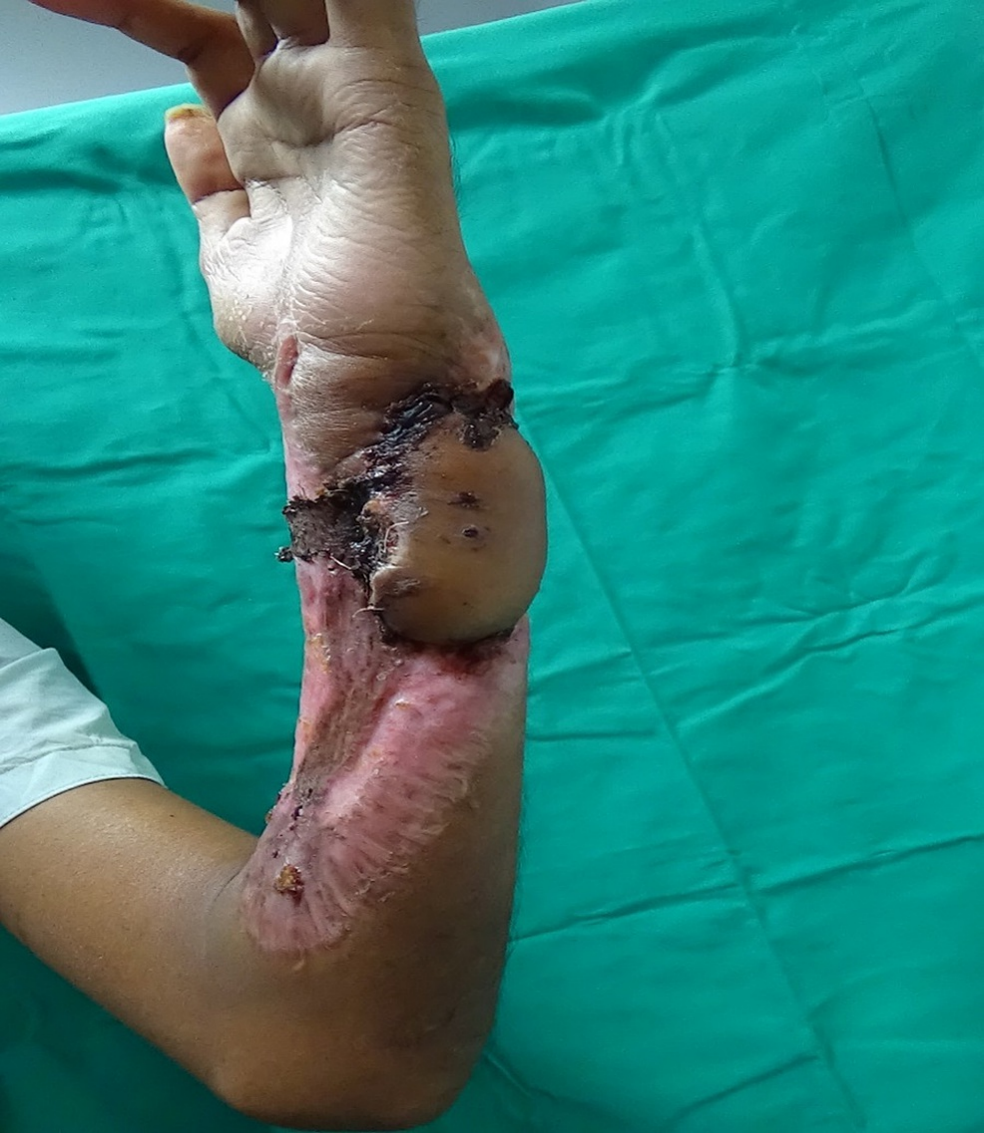
The appearance of the flap covering the ulnar bone defect three months post-operation.

He underwent soft tissue coverage with a left pedicled groin flap for the area of exposed ulnar bone. The rest of the wound was managed by dressing. On day 21 post-operation, the flap was divided, and the underneath raw areas were grafted with a split-thickness skin graft. Three months post-operation, the flap appeared bulky; however, there was no residual area of defect or exposed bone, as shown in Figure 6.

## Discussion

Filatov, Ganzer, and Gillies were the earliest surgeons to apply the tubed pedicle flap and were reported in the literature in 1917 [2]. The skin flap was taken from the thorax as a bipedicle and used to reconstruct an extensive facial skin defect [3]. As a result, this concept was popularized by many surgeons. For example, Bakamjian in 1965 [5] popularized the deltopectoral flap for pharyngoesophageal reconstruction, and McGregor in 1972 [1] popularized the groin flap for reconstruction of the upper extremities. These pedicle flaps were based on a single pedicle and were involved in two-stage procedures. After the flap is raised and inset, the flap will be divided 21 days later.

Although the distant tubed pedicle flap has been used for nearly a century, it is still widely used in reconstruction, particularly in upper limb defects [4]. Pedicled flaps from the groin and abdomen were the workhorses for hand and forearm reconstruction in the last three decades, yet they are still relevant now. Many plastic surgeons have preferred them because of the high flap survival from the excellent blood supply and because they are technically friendly [6]. They are generally used in upper extremity defects with an extensive zone of injury in the recipient's vessels, such as full-thickness burns involving the upper limbs, as mentioned above. Soft tissue coverage with a free flap in this kind of injury has a greater chance of anastomotic thrombosis and flap necrosis [3].

The pedicled groin flap that McGregor introduced in 1972 has several advantages over other pedicled flaps. First, the inconspicuous donor site gives a better cosmetic perception, especially to women. For example, as in the deltopectoral flap, the donor site may have a large scar in the upper chest. Second, the distal portion of the groin flap, which is the part set into the recipient site, is usually hairless, even in hirsute individuals. Third, the groin flap appears less bulky than the abdominal flap since it has thinner subcutaneous tissue. Last, tissue edema is less likely to occur because of the excellent venous drainage at its base [7].

Pedicled flaps have many disadvantages, like being uncomfortable for the patients as they have to keep the limb attached to the flap for 21 days, their bulkiness, and the need for multiple stages of surgery [8]. In our case, we plastered the limb to the torso with an adhesive fabric sheet, giving the patient more freedom to move the arm, and shoulder stiffness could be avoided. However, the movement must not compromise the flap since we have narrowed the base. In addition, because of its axial blood flow and a length-to-base ratio about three times greater than the classic abdominal flap, this will allow more freedom of movement of the fingers [7]. Some surgical improvements have overcome these disadvantages, especially limb movement, and flap bulkiness. Some surgeons keep the base narrow to tube the flap comfortably and give more mobility to the limb [8]. To avoid flap bulkiness, fat at the skin edge can also be trimmed and beveled for ease of inset and a better cosmetic appearance. The bulky flap can also be thinned during the secondary procedure until the subdermal level [9]. Surgeons may perform micro-dissection of the deep branch of the superficial circumflex femoral system, allowing the flap to be raised extremely thin and larger, resulting in a more favorable cosmetic result.

One of the major downsides of a pedicled flap is its lack of sensation. This is particularly disabling to a patient if both the thumb and fingers are involved. To overcome this, patients may require subsequent microsurgical procedures like wrap-around flaps or plantar sensated skin transfers [8].

## Conclusions

Despite the fact that the pedicle-tubed groin flap is considered one of the old fashions for soft tissue coverage, many surgeons believe it is still relevant and practically used for reconstruction in upper limb trauma instead of the more popular free tissue transfer because it is versatile, has less demand on infrastructure, and is cost-effective. In addition, it is generally an efficient method and still able to achieve the aim of reconstruction with a good coverage outcome.

## References

[1] McGregor IA, Jackson IT (1972). The groin flap. Br J Plast Surg.

[2] Webster JP (1959). The early history of the tubed pedicle flap. Surg Clin North Am.

[3] Climo MS (1978). Split groin flap. Ann Plast Surg.

[4] Al-Qattan MM, Al-Qattan AM (2016). Defining the indications of pedicled groin and abdominal flaps in hand reconstruction in the current microsurgery era. J Hand Surg Am.

[5] Bakamjian VY (1965). A two-stage method for pharyngoesophageal reconstruction with a primary pectoral skin flap. Plast Reconstr Surg.

[6] Chuang DC, Colony LH, Chen HC, Wei FC (1989). Groin flap design and versatility. Plast Reconstr Surg.

[7] Knutson GH (1977). The groin flap: a new technique to repair traumatic tissue defects. Can Med Assoc J.

[8] Sabapathy SR, Venkatramani H, Martin Playa P (2015). The use of pedicled abdominal flaps for coverage of acute bilateral circumferential degloving injuries of the hand. Trauma Case Rep.

[9] Sabapathy SR, Bajantri B (2014). Indications, selection, and use of distant pedicled flap for upper limb reconstruction. Hand Clin.

